# Barriers and Facilitators Associated With App-Based Treatment for Female Urinary Incontinence: Mixed Methods Evaluation

**DOI:** 10.2196/25878

**Published:** 2021-09-17

**Authors:** Nienke J Wessels, Anne M M Loohuis, Henk van der Worp, Linde Abbenhuis, Janny Dekker, Marjolein Y Berger, Julia E W C van Gemert-Pijnen, Marco H Blanker

**Affiliations:** 1 Department of General Practice and Elderly Care Medicine University Medical Centre Groningen University of Groningen Groningen Netherlands; 2 Center for eHealth Research and Disease Management Faculty of Behavioural Sciences University of Twente Enschede Netherlands

**Keywords:** mHealth, female, mixed methods, primary health care, urinary incontinence

## Abstract

**Background:**

App-based treatment for urinary incontinence is a proven effective and cost-effective alternative to care as usual, but successful implementation requires that we identify and address the barriers and facilitators associated with app use.

**Objective:**

The goal of the research was to explore the factors influencing app-based treatment for urinary incontinence and identify which barriers or facilitators are associated with treatment success or failure.

**Methods:**

We used a sequential explanatory mixed methods design to connect the results of a randomized controlled trial with data from semistructured interviews. This previous RCT had shown the noninferiority of app-based treatment compared with care as usual for urinary incontinence over 4 months. Participants who reported success or failure with app-based treatment, as measured by the change in International Consultation on Incontinence Modular Questionnaire Urinary Incontinence Short Form symptom score, were selected for telephone interview by purposive sampling (n=17). This study reports mainly on the qualitative component of our mixed methods study. Qualitative analyses were conducted in two ways. First, we analyzed the qualitative data of all interviewed participants and discussed the relationships between the main themes. Second, the experiences between the success (n=9) and failure group (n=8) were compared and contrasted to explore factors that were positively or negatively associated with the quantitative effect of app-based treatment. These factors were then interpreted as barriers to and facilitators of successful app-based treatment.

**Results:**

Four interrelated themes were identified as affecting the app based treatment effect: adherence, personal factors, app factors, and awareness. Qualitative analyses of the relationships between the themes showed that adherence-related factors directly influenced treatment effect in both a positive and negative matter. In turn, adherence was also positively and negatively influenced by the other 3 themes. Additionally, awareness was positively influenced by the treatment effect. Within these themes, several factors were identified that acted as barriers (eg, unrealistic expectation of time investment and interfering personal circumstances), facilitators (eg, strict integration of exercises and prior pelvic floor muscle therapy), or both (eg, personality traits and increased awareness of symptoms).

**Conclusions:**

This study shows that the effect of app-based treatment for urinary incontinence is mainly influenced by adherence, which in turn is affected by personal factors, app-based factors, and awareness. The identified factors could function as both facilitators and barriers depending on the user and interaction with other themes. Insight into these facilitators and barriers could lead to improved implementation and increased treatment effectiveness by targeting women most likely to benefit and through further development of the app.

**International Registered Report Identifier (IRRID):**

RR2-10.1002/nau.23507

## Introduction

The use of mobile health (mHealth) for urinary incontinence can be an effective and cost-effective alternative to care as usual [1-3]. Although implementation must now proceed for us to realize these benefits for patients and caregivers, successful uptake of the app requires that we identify and address the barriers and facilitators associated with this treatment modality [4]. The complexity of mHealth interventions with multiple interacting components calls for a thorough evaluation of the connection between patient experience, adherence, and effectiveness but is often lacking [5,6]. Previous qualitative studies of women suffering from urinary incontinence have identified factors that could affect app- or internet-based treatment for urinary incontinence by exploring their expectations and experiences [7-10]. Women expected that internet-based treatment would be more accessible, more flexible, and improve treatment adherence, but they expressed concern about the lack of contact with a caregiver [7,8]. Only two studies focused specifically on user experiences with mHealth for urinary incontinence over periods of 6 weeks to 3 months [9,10]. Women commented on several positive and negative effects: support via reminders, insecurity of the treatment result, and increased awareness of symptoms [9,10]. However, these experiences with internet- or app-based treatment for urinary incontinence were never assessed in relation to quantitative treatment success or failure. It is important to explore if a relation exists between the factors identified in qualitative research and the actual success or failure of the intervention [11]. This could reveal strategies for tailoring the app, increasing its effects, or targeting women most likely to benefit.

In this study, we aimed to explore the factors influencing app-based treatment for urinary incontinence and identify which barriers or facilitators are associated with treatment success or failure.

## Methods

### Study Design

**Figure 1:**
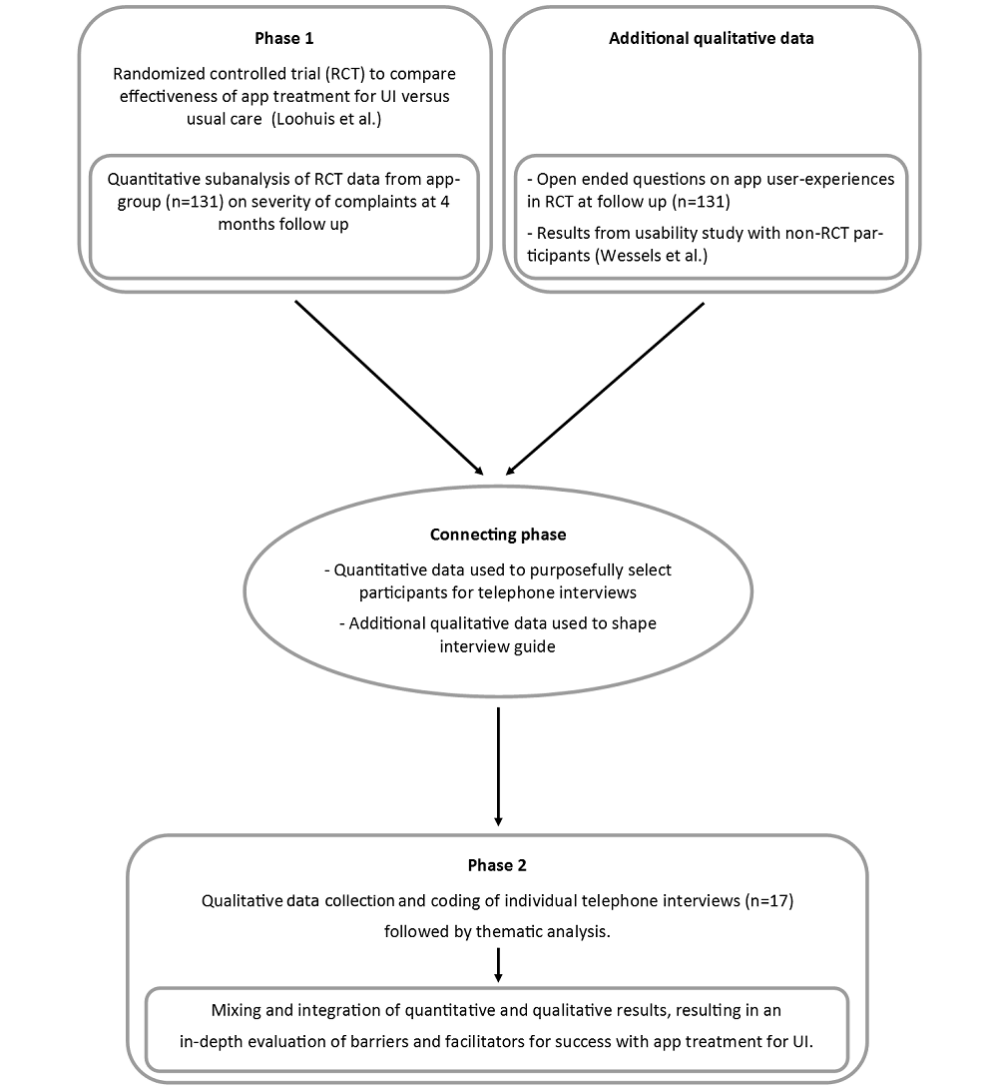
Description of sequential explanatory mixed methods study to explore barriers and facilitators for success with app treatment for urinary incontinence.

We conducted a mixed methods study with a sequential explanatory design that built on a previously reported quantitative randomized controlled trial (RCT) by integrating the results of a qualitative analysis of interviews. The qualitative phase reported in this manuscript follows from a quantitative phase that was reported elsewhere [3,12] (Multimedia Appendix 1) and links both phases in a connecting phase (Figure 1). The original RCT showed the noninferiority of app-based treatment for urinary incontinence (containing a step-by-step self-management program based on Dutch general practitioner and international guidelines [13,14]) compared with care as usual after 4 months [3], and for this study we used the quantitative outcomes of the URinControl RCT to select participants for telephone interview by purposive sampling. The qualitative results from the interviews were expected to refine and explain the quantitative results by exploring participants’ views in more depth [11,15,16]. The Research Ethics Committee (no. M17.207954) and Medical Ethical Review Board (METc-no.: 2014/574) of the University Medical Center Groningen, Netherlands, approved the study. All participants gave written informed consent.

### Participants

We used purposive sampling to select women for interview from the app-based treatment group according to the change in symptom severity measured by the International Consultation on Incontinence Modular Questionnaire Urinary Incontinence Short Form (ICIQ-UI-SF) at 4 months [17]. We ranked the change in ICIQ-UI-SF score from the largest increase to the largest decrease in symptoms and invited participants by working inward from these extremes. In this way, we created two groups: a treatment success group and a treatment failure group. We approached women who had completed the 12-month follow-up requirement to avoid influencing the ongoing RCT. Women were invited by telephone, after which an appointment was made for a telephone interview.

### Data Collection

The semistructured interview guide contained several broad themes to ensure that all relevant topics were covered in the interviews. These were selected based on a literature review and the results of a study of the experiences of URinControl app users not included in the RCT [10]. We also reviewed the answers to open-ended questions regarding the experiences of all users in the app group to help further shape the interview guide (see connecting stage in Figure 1).

A female medical master’s student (LA) who had no prior relationship with the participants conducted telephone interviews in April and May 2019. She is experienced in performing in-person interviews and prepared for the current task by conducting an extensive literature review on this subject. Additionally, we held a pilot interview and regular peer debriefings to evaluate the quality of the interviews. The interviewer encouraged participants to elaborate on their experiences and asked them to raise any subject they felt relevant that had not yet been covered. Interviews where audio recorded and transcribed verbatim.

### Data Analysis

Qualitative analysis was driven by an inductive approach, allowing new patterns and categories to emerge from the raw data. Interview transcripts were coded separately by two researchers (NW, LA) using Atlas.ti (version 8.4, Atlas.ti Scientific Software Development GmbH), and the codes and emerging categories were compared and checked for consensus. Additionally, we regularly discussed broader themes emerging from the categories within the research group and compared the raw data to ensure that the themes covered all aspects. Interviews were conducted until saturation (no new categories emerged in 3 consecutive interviews). Analysis then proceeded in 2 stages. First, we focused on the coded data of all interviewed participants and discussed the relationships between the main themes. Second, we integrated the quantitative and qualitative data by comparing and contrasting the experiences between the success and failure groups, describing the between-group differences in subthemes and the relations between main themes. Additionally, between-group differences in subthemes were checked by frequency counts. Multimedia Appendix 2 and 3 provide a more detailed description of the qualitative analysis and the coding tree.

The descriptive analysis of participant characteristics was conducted with SPSS (version 26, IBM Corp). Reporting was in accordance with the Consolidated Criteria for Reporting Qualitative Research (COREQ) [18].

## Results

### Participant Selection and Characteristics

**Figure 2:**
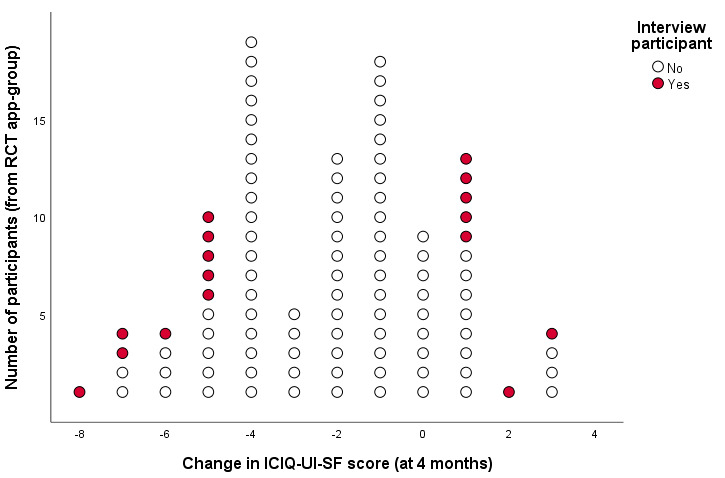
Overview of the interview participants (n=17) with respect to the total randomized controlled trial app group (n=102). International Consultation on Incontinence Modular Questionnaire Urinary Incontinence Short Form change scores: negative scores indicate symptom improvement (success) and positive scores indicate symptoms increasing (failure).

The change in urinary incontinence severity measured with the ICIQ-UI-SF in the 102 women with complete follow-up at 4 months ranged from –8 to +3 points (mean 2.2, SD 2.56; Figure 2). As mentioned, women were invited by working inward from the largest increase and largest decrease in the change in ICIQ-UI-SF score. Three women had not completed 12 months of follow-up and were not invited, and 5 women declined the invitation to participate. Data saturation was reached after the 17th interview. The interviewed women were aged 35 to 78 years and had suffered from urinary incontinence for between 3 months and 20 years (Table 1).

**Table 1:** Characteristics of the interview participants^a^.

Participants					UI^b^ outcomes			UI at baseline					Relevant experience	
	#	Age (years)	Level of education^c^	Severity^d^ change		PGI-I^e^	Severity score		Impact^f^ score	Type	Duration (years)	Previous PFMT^g^		Smartphone/tablet^h^ (years)
**Treatment success**														
	1	65	Higher	–6		6	10		33	Stress	4	No		8
	2	67	Higher	–8		6	16		59	Urge	5	No		2
	3	54	Lower	–7		7	12		42	Stress	20	Yes		2
	4	61	Lower	–5		6	10		37	Stress	20	Yes		5
	5	46	Higher	–5		6	7		32	Stress	2	No		8
	6	48	Higher	–7		5	13		36	Stress	6	No		15
	7	71	Higher	–5		5	14		51	Urge	15	No		8
	8	78	Lower	–5		6	10		37	Stress	20	Yes		1
	9	44	Lower	–5		5	11		32	Urge	15	No		—^i^
**Treatment failure**														
	10	54	Lower	2		6	6		32	Stress	5	No		5
	11	65	Lower	3		4	5		23	Stress	10	No		6
	12	48	Lower	1		6	12		51	Stress	12	Yes		10
	13	48	Higher	1		5	4		25	Urge	16	No		10
	14	43	Higher	1		4	5		27	Urge	0.25	No		3
	15	63	Higher	1		4	4		32	Urge	3	No		7
	16	42	Higher	1		6	7		26	Stress	0.42	No		6
	17	35	Lower	1		5	9		27	Urge	20	Yes		—

^a^Women using app-based treatment purposefully sampled based on change of urinary incontinence severity (ICIQ-UI-SF score) after 4 months. All measures were self-reported and recorded at baseline except for the ICIQ-UI-SF change score and the PGI-I, which were recorded at 4-month follow-up.

^b^UI: urinary incontinence.

^c^Lower: primary or secondary education; higher: tertiary education or higher.

^d^Severity was based on International Consultation on Incontinence Modular Questionnaire Urinary Incontinence Short Form; range 0-21, higher score means worse incontinence.

^e^PGI-I: Patient Global Impression of Improvement, Likert scale ranging from 0 (very much worse) to 7 (very much better), with 4 reflecting no change.

^f^Impact based on International Consultation on Incontinence Modular Questionnaire Lower Urinary Tract Symptoms Quality of Life; range 19-67, higher score reflects larger impact of urinary incontinence on quality of life.

^g^PFMT: pelvic floor muscle therapy.

^h^Years in possession of device.

^i^Not applicable.

Overall, 9 women experienced treatment success and 8 experienced treatment failure (Figure 2). The change in ICIQ-UI-SF score for women from the success group ranged from –8 to –5 points (median –5.5); for the failure group, it ranged from 1 to 3 (median 1.5). Patients from the success group seemed to have worse urinary incontinence–specific measures and higher age at baseline. Educational level and relevant experiences seemed comparable between groups. None of the patients from the failure group experienced a worsening of symptoms (Patient Global Impression of Improvement [PGI-I] <4).

### Semistructured Interviews

#### Main Themes

**Figure 3:**
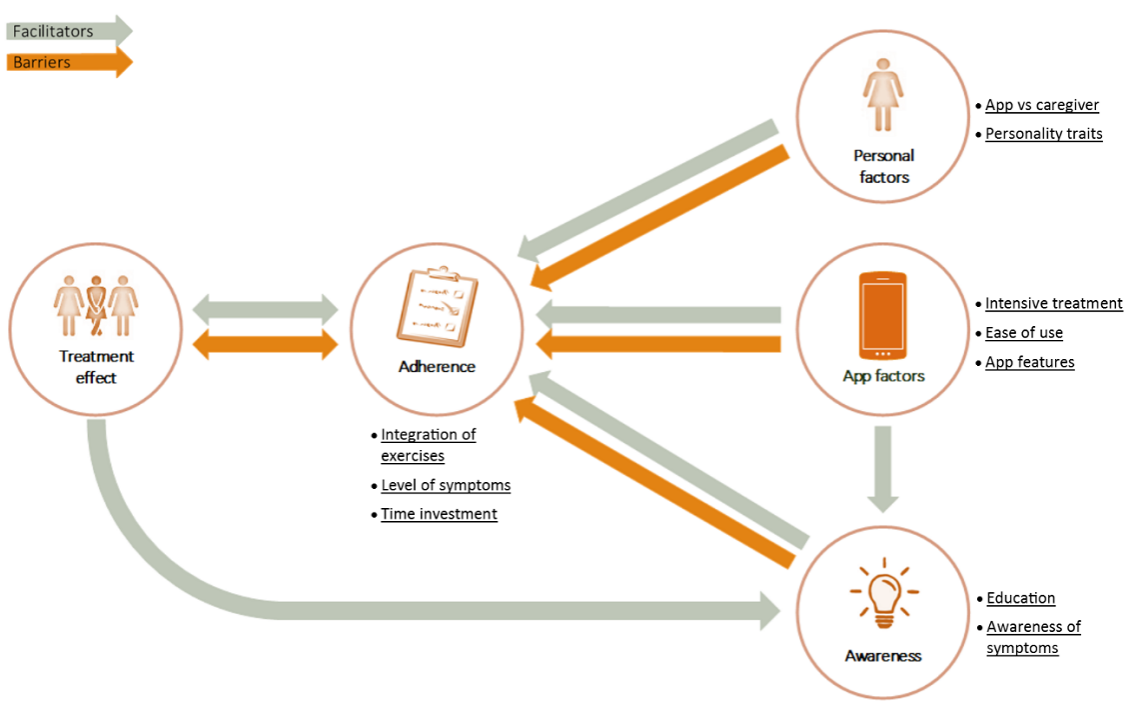
Cross-thematic network of interrelated themes resulting from the qualitative analysis of telephone interviews (n=17). Subthemes show the barriers or facilitators for successful app treatment.

We identified adherence, personal factors, app factors, and awareness as the main themes related to overall treatment effect. Discussion of the relationships between the themes resulted in a cross-thematic network (Figure 3). Factors in the adherence theme directly influenced app-based treatment effects as a barrier and facilitator. Adherence was further influenced by factors in the personal factors, app factors, and awareness themes (barriers and facilitators). Finally, awareness was facilitated by the treatment effect and by app factors.

There were no differences between the success and failure groups in the main themes or relationship directions, but there were differences between those groups in the subthemes and in the strength of the relationships between the main themes. The frequency counts for quotes showed between-group differences in subthemes with clear patterns that matched those found in the interviews (Table 2).

**Table 2:** Themes and subthemes by treatment success and failure^a^.

Theme and subtheme					Groups		
					Success group		Failure group
**Personal factors**							
	**App versus caregiver**				—^b^		—
		Prior pelvic floor muscle therapy		3		—	
		Being independent of care provider		3		4	
		Insecure about correctly performing exercises		2		1	
		Lowering shame barriers		2		—	
	**Personality traits**				—		—
		Positive (eg, go-getter, disciplined)		2		—	
		Negative (eg, slacking off)		2		4	
**App factors**							
	Intensive treatment				3		2
	**Ease of use**				—		—
		**Devices**		—		—	
			Tablet	3		3	
			Smartphone	6		6	
		Complex user interface		3		2	
		**Lessons (exercise levels)**		—		—	
			Useful	7		5	
			Not useful	1		1	
	**App features**				—		—
		**Reminders**		—		—	
			Useful	4		3	
			Not useful	6		2	
			Timing inconvenient	3		4	
		**Graphs**		—		—	
			Useful	3		1	
			Not useful	5		7	
**Awareness**							
	Education				5		3
	**Awareness of symptoms**				—		—
		Positive		9		5	
		Negative		—		2	
**Adherence**							
	Integration of exercises				4		6
	**Level of symptoms**				—		—
		Recurrence of symptoms (positive)		5		1	
		Improvement of symptoms (positive)		1		—	
	**Time investment (negative)**				—		2
		Personal circumstances (negative)		3		5	

^a^Numbers are representative of how many participants mentioned the subtheme throughout the interviews.

^b^Not applicable.

#### Adherence

##### General Findings

The adherence theme covered factors affecting the level to which participants felt they adhered to treatment advice (ie, app use and performing exercises). Women in both groups felt that their adherence was directly related to the treatment effect, and it was evident that increasing and decreasing symptoms each affected their motivation to adhere to treatment. Several subthemes emerged.

##### Integration of Exercises

The intensive treatment and frequent reminders provided by the app helped women perform exercises at set times. This enabled them to establish exercise routines that suited their schedules and contributed to the overall treatment effect women experienced. Women in the failure group tended to describe less strict exercise regimes than women in the success group.

After a while you’ll get into a certain rhythm. I did it on my way to work.... those are the moments you remember, so you get a regularity to it. That is pretty nice.

P6, success

Or when I’m in the car, I have nothing to do, and I’m bored; during a long drive, for example. They are the kind of exercises you can do everywhere with no one taking notice.

P10, failure

##### Level of Symptoms

Women in both groups mentioned that symptom severity influenced adherence, both positively and negatively. One woman in the failure group stated that her low symptom level meant that she lacked the motivation to persevere with the exercises, resulting in minimal treatment effect. Women in the success group more often stated that both symptom improvement and symptom recurrence after a period of less adherence motivated them to start again, hereby enhancing their training results.

No, I think that my complaints need to be more severe for that [increased adherence]. Now I just think “I’ll use a pantyliner and I’ll be done with it.”

P15, failure

…That’s why I keep doing it. Because I stopped for about three weeks, because I had surgery on my foot and was hospitalized for it. But after that I could notice that I hadn’t done it.

P8, success

##### Time Investment

Some women in the failure group felt that the treatment program was much more time consuming than expected, which markedly decreased their adherence and limited their treatment effect. A lack of time due to personal circumstances such as illness, family reasons, or life events also negatively influenced adherence in both groups, but this was mentioned more by women in the failure group.

#### Personal Factors

This theme covers personality traits and attitudes toward app-based treatment in comparison with treatment by a care provider.

##### App Versus Caregiver

Women in both groups valued the concept of 24-hour treatment availability and liked being independent of a care provider. This enabled them to be in control of their own treatment and combine it with their busy and irregular lifestyles, which made it easier for them to adhere to the treatment.

Great [opinion about being randomized in app group], because in all honesty, I wasn’t looking forward to it at all. I thought, then I’ll have to go a general practitioner and make another appointment again. But with an app, you are the one in control, which is much easier.

P15, failure

A few women mentioned they occasionally wondered if they performed the exercises correctly, and although none had consulted a care provider, they stated that they might do so in the future. For some women in the success group, preference for the app arose from having experienced insufficient results from prior physical therapy for incontinence. Additionally, one woman stated that she preferred the app because she felt a major barrier when talking about her symptoms.

I thought it would be very convenient to try the app, because this [urinary incontinence] is not something I would easily consult my general practitioner for. … It’s just not something people talk about.

P1, success

##### Personality Traits

Personal characteristics were frequently mentioned as barriers or facilitators of success. Women in the success group mainly declared it was a matter of just doing it and being a bit of a go-getter to continue with the exercises on a regular basis. Conversely, women in the failure group tended to focus on negative traits and described knowing themselves as sloppy and not being able to persevere, which negatively impacted their adherence.

It’s just a matter of carrying on, and you’ll start getting results.

P3, success

That is the same as going to the dentist and thinking, maybe I should brush my teeth thoroughly for a change [laughs]. ... It’s not so much the app having to change, I think it [low adherence] is something engrained in humans.

P12, failure

#### App Factors

Subthemes related to app factors (ie, experiences with different features) included the intensity and extensiveness of treatment, ease of use of the app, and features within the app.

##### Intensive Treatment

Women in both groups appreciated the intensive and extensive treatment program offered by the app, indicating that they felt this was something a caregiver could not provide.

...I think you are more dedicated to it, especially at the start. When you go to the physical therapist, you get some exercises, You go home, and you do those. But with the app, you just do it every day.

P4, success

##### Ease of Use

Most women installed the app on their smart phone because this device was always at hand, which made it easier to adhere to the intensive treatment. Others preferred a tablet because of the larger display or because they did not possess a smartphone or know how to operate one. Most women in both groups found the app easy to use and appreciated the clear instruction provided in the lessons. However, a few women stated that the app’s user interface was overly complex, taking too long to identify where to start and how to get an overview of the content. This negatively impacted their motivation to use the app.

##### App Features

Most women tried the app’s reminder function, but it yielded mixed feelings regarding the effect on their treatment adherence. Despite being able to set 3 reminders per day, many women in both groups found the timings inconvenient or did not want to receive a reminder when they were with other people. Also, some women were unaware that the app provided this function. Overall, despite many women appreciating the inclusion of a reminder function, a slightly larger cohort (mainly from the success group) stated that they ultimately stopped using this feature.

No, I felt those [reminders] were actually only annoying because I already had my own vision of when I was generally going to practice.

P12, failure

I was planning on doing them only when I was by myself. I did not want to receive a reminder when I was out somewhere.

P1, success

Some women stated that the graphs provided insights about progression and made them more aware of their symptoms, but only a few participants used and appreciated this function. When used, the function did give a sense of being on the right path and encouraged perseverance. One participant from each group stated they thought it would have been more motivational to have a graphical display showing symptom changes. However, many women, mostly from the failure group, found that the graphs were difficult to interpret or that they added little. One woman declared that she found looking at the graphs to be too confrontational.

I would leave that out; when you’ve practiced and all the statistics. If you skipped that for a day, you’ll start feeling guilty.

P16, failure

#### Awareness

##### General Findings

There was increased awareness in several domains. Awareness increased concerning knowledge of the disease (education) and awareness of symptoms. This increased awareness could act as either a facilitator of or a barrier to adherence. Furthermore, awareness increased directly with both app factors (reminders) and treatment effects (symptom improvement or recurrence).

##### Education

Women in both groups found the information provided by the app useful. Many had thought urinary incontinence was a part of life they had to accept. Knowledge about the possible effect of conservative therapies enhanced their motivation to carry on with the exercise program. Others stated that they felt less alone dealing with urinary incontinence knowing that other women experienced the same symptoms.

##### Awareness of Symptoms

The intensive treatment resulted in increased awareness of the impact of symptoms and of coping strategies used. In general, women in both groups appreciated this aspect. In the failure group, women stated that they liked knowing what to do to improve their symptoms. In the success group, women tended to report putting this knowledge into action, stating several key benefits: they felt more confidence in their treatment and the treatment helped them to make lifestyle changes and lessened the sense of taboo when talking with other women about symptoms.

Well, I certainly know what to do now to get results. I know that I have to do it for months, then it will work. That I understand.

P11, failure

It gave me some reassurance in the sense of “that should be doable.” And that already made it easier to postpone toilet visits.

P7, success

Conversely, a few women in the failure group stated that they did not like the increased focus on themselves and their problems, which made them less motivated to use the app. One even wondered if this had led to her symptoms increasing.

## Discussion

### Principal Findings

Our findings provide new insights into the barriers and facilitators associated with successful app-based treatment for urinary incontinence. Additionally, this study contributes to the growing understanding of barriers or facilitating factors influencing mHealth use and ways to overcome or improve them [6].

Our results principally show that the effect of each explored factor results from whether there is treatment success or failure. Moreover, the views of patients concerning adherence to app use and performing the recommended exercises were key. Comparison between the success and failure group revealed several factors that facilitated treatment success, namely strict integration of exercises, previous experience of face-to-face pelvic floor muscle therapy (PFMT) with insufficient effect, and being a so-called go-getter; by contrast, we identified the barriers as being unrealistic expectations of time investment, interfering personal circumstances, and being unable to persevere. Of note, however, the graphs and reminder functions did not have the expected facilitating effect and, indeed, sometimes acted as a barrier. It was interesting that the general increased awareness after treatment and the awareness of symptom change positively and negatively affected adherence and treatment effectiveness. We believe these facilitators and barriers can be used to improve outcomes with app-based therapy.

### Strengths and Limitations

This is the first study using a sequential explanatory design to assess the facilitators of and barriers to app-based treatment for urinary incontinence. We consider the mixing and integration of qualitative and quantitative data throughout the study to be an important strength, helping to improve the quality of our conclusions [12]. This approach produces a whole that is greater than the sum of the individual qualitative and quantitative parts [19]. We also selected high- and low-performing cases to explore the contrast between treatment success and failure [11], which enabled us to identify facilitators and barriers associated with the desired treatment effect. Other strengths of our design were the use of previously collected qualitative data to build the interview guide, the reevaluation of themes within each outcome group, and the use of quote frequency counts.

Despite these notable strengths, however, there were some important limitations. For example, there was no member check due to logistic difficulties, and 8 women were unavailable for interview, potentially affecting the identified themes. Additionally, it should be noted that the exploratory nature of this type of (qualitative) research allows for hypothesis generation, not hypothesis testing. Therefore, when interpreting the results, one should keep in mind that this research is not able and does not seek to predict treatment effect. This research rather explores the factors influencing treatment from a qualitative participant perspective and relates these to the quantitative treatment effects.

Participant selection for the interviews was based on follow-up outcomes at 4 months, which we anticipated would reflect the optimum treatment effect. However, interviews were postponed until after the 12-month follow-up to limit interference with the trial. Although this extension allowed us to explore facilitators and barriers in both the short- and long-term, it could have introduced recall bias in the women’s experiences and perception of factors influencing effectiveness in the first 4 months.

There was also some inconsistency with the concepts of failure and success. Among the women with a deterioration in urinary incontinence severity on the ICIQ-UI-SF at 4 months, none perceived a worsening on the PGI-I at that time and none reported treatment failure in the interviews after 12 months. Recall bias could explain the inconsistency between the ICIQ-UI-SF at 4 months and the interview after 12 months but not the difference between the PGI-I and the ICIQ-UI-SF, both of which were measured at 4 months. Thus, it may be that these differences indicate that the perception of improvement reflects not only the change in urinary incontinence symptoms but also better coping strategies or decreased shame due to increased knowledge.

### Comparison With Existing Literature

Previous studies have included women with no experience with eHealth for urinary incontinence, using eHealth for urinary incontinence for 6 weeks to 3 months, and with no case selection based on treatment effect [7-10]. In this study, we explored the experiences of women using the app for 12 months who had showed a clear worsening or improvement of symptoms. Although we identified similar main themes, we could also further explore the relationship between those themes and the treatment effect. Consistent with existing research, women from both of our study groups expressed positive views about the availability, flexibility, privacy, and education provided by eHealth for urinary incontinence [7-10].

#### Insecurity About Exercise Performance

Women’s feelings in our study were mixed with regard to insecurities about the correct performance of exercises. Firet et al [7] described that women had experienced their pelvic floor muscles to be difficult to contract correctly during face-to-face PFMT. Elsewhere, Asklund et al [9] reported that a lack of reassurance created insecurity when women thought contractions were good enough but were left wondering if personal instruction could lead to improvements. In our study, women expressed these insecurities in both the success and failure groups, but despite being instructed to consult a health care professional if they needed, none sought further advice. This suggests that the presence of insecurity about treatment is not a differentiating factor for treatment success or failure. Instead, treatment failure in women with insecurities may have reflected other barriers (eg, not being able to persevere or having interfering personal circumstances) or different coping strategies, with insecurities and doubts keeping them from consulting a caregiver.

Awareness of urinary incontinence symptoms and treatment options acted as both a facilitator and a barrier for women in our study, whereas in other studies, increased awareness was mainly described positively [8,9].

#### Increased Awareness: Positive Effects

Positive effects found in our study were an increased awareness of symptoms and treatment options, which lessened the sense of taboo around the topic and encouraged women to change their lifestyles. Additionally we confirmed that awareness of symptom recurrence after a period of lower adherence stimulated motivation, as Asklund et al [9] also described.

#### Increased Awareness: Negative Effects

Negative effects were related to a negative focus on symptoms and a decrease in adherence to treatment. The increased negative focus on symptoms in some women acted as a barrier as it kept them from continuing app use, which was also reported by Wessels et al [10]. Also, for some women, awareness of symptom improvement during treatment led to decreased motivation to adherence to the treatment.

#### App Features

Additionally, it was notable that reminders did not facilitate treatment success and the graph function was deemed too confrontational or unhelpful, contrasting with our expectation that these would positively affect motivation and adherence [9,10]. This may be related to the long 12-month follow-up period. For example, the facilitating effect of reminders may have been small or only present early on, potentially being lost due to recall bias. The sense that the graphs were confrontational may have appeared over time in response to a lack of treatment effect, but this may also have resulted because the graphs only monitored lack of adherence, rather than progress or change in urinary incontinence symptoms. Asklund et al [9] showed the same statistics in their graph use but did not report this confrontational effect.

### Implications for Research and Practice

The findings of our study can be used to increase the effect of app-based treatment by targeting women who are most likely to benefit and showing how we can better tailor app-based treatments. When the app is made available to the wider public, it will be important to inform potential users about the various factors that can influence the treatment effect. When care providers discuss the use of app-based treatment for a patient with urinary incontinence, our findings indicate it is crucial they consider personality traits (eg, highly self-motivated), expectations of time investment, and previous experiences with regular PFMT. We can tailor the app-based treatment to increase the treatment effect by modifying the graph and reminder functions. Graphs could be an optional tool that are simplified to emphasize urinary incontinence symptom progression rather than lack of adherence. To reduce the perceived intrusion of the reminder function, this could be revised to a daily to-do list with no preset times. Finally, future research could be focused on further examining the characteristics of women in whom app-based treatment failed because this might be a distinct group with similar personality traits. This knowledge could help health care professionals provide the necessary support for patients to achieve treatment success.

In conclusion, this study shows that the effect of app-based treatment for urinary incontinence is mainly influenced by adherence, which in turn is affected by personal factors, app-based factors, and awareness. However, it was notable that the identified factors could function as both facilitators and barriers depending on the user and the interaction with other factors. Insight into these facilitators and barriers can be used to increase the treatment effect of app-based treatment for urinary incontinence by ensuring that we target women most likely to benefit. Introducing some minor changes to the graph and reminder functions could improve the usability of our app.

## Appendix

Multimedia Appendix 1 Design summary.

Multimedia Appendix 2 Supplemental methods.

Multimedia Appendix 3 Coding tree including major topics and subthemes.

## Abbreviations

COREQ: Consolidated Criteria for Reporting Qualitative Research
ICIQ-UI-SF: International Consultation on Incontinence Modular Questionnaire Urinary Incontinence Short Form
mHealth: mobile health
PFMT: pelvic floor muscle therapy
PGI-I: Patient Global Impression of Improvement
RCT: randomized controlled trial
