# Supplementary material for: Barriers and Facilitators Associated With App-Based Treatment for Female Urinary Incontinence: Mixed Methods Evaluation
Source: JMIR Mhealth Uhealth. 2021 Sep 17;9(9):e25878. doi: 10.2196/25878 (PMC8486988; doi:10.2196/25878)
Supplement: Multimedia Appendix 1 [file mhealth_v9i9e25878_app1.docx]

*Randomized controlled trial:*

From July 2015 through July 2018, we recruited 262 women for the RCT in the north of the Netherlands. Women were recruited through general practitioners, social media, and the lay press. The following inclusion criteria were used: female sex; age ≥18 years; self-reported stress, urgency, or mixed UI at least twice a week according to the Three Incontinence Questions (3IQ); wanting treatment; and access to a smartphone or tablet. Women with the following were excluded: indwelling urinary catheter, urogenital malignancy, previous surgery for UI, treatment for UI in the previous year (pharmacological or non-pharmacological), terminal or serious illness, cognitive impairment, psychiatric illness, urinary tract infection (dipstick, and if negative, dipslide or urine culture), overflow or continuous UI, pregnancy or recent childbirth (<6 months ago), or the inability to complete a questionnaire in Dutch.

After randomization, women in the intervention group received instructions to install the URinControl app on a smartphone or tablet. The app contained a step-by-step program for the self-management of UI based on Dutch GP and international guidelines for treatment of UI.^11,12^ It provided information about UI, lifestyle advice, exercises to increase awareness of the pelvic floor muscles, and exercises for pelvic floor muscle therapy (PFMT) and bladder training. Depending on the type of UI identified, instructions within the app directed the user to relevant information and exercises. The app also provided reminders and graphical feedback of the number and level of exercises performed. Additional information on the development and content of this app has been reported previously.^12^ Participants were free to contact their GP with any questions regarding medical aspects and/or to receive additional treatment.
