# Supplementary material for: Barriers and Facilitators Associated With App-Based Treatment for Female Urinary Incontinence: Mixed Methods Evaluation
Source: JMIR Mhealth Uhealth. 2021 Sep 17;9(9):e25878. doi: 10.2196/25878 (PMC8486988; doi:10.2196/25878)
Supplement: Multimedia Appendix 2 [file mhealth_v9i9e25878_app2.docx]

*Process of qualitative data analysis.*

Transcripts of the telephone interviews were coded separately by two researchers (NW, LA). A consensus check was performed by NW after coding of the first 3 transcripts, showing the use of similar codes throughout the transcript. Minor adjustments to the coding tree were made accordingly. Coding continued separately and emerging new categories were regularly discussed within the research group. This was driven by an inductive approach, allowing new patterns and categories to emerge from the raw data. Interviews were conducted until no new categories emerged in three consecutive interviews. (i.e., until we had reached saturation). Broader themes emerging from the categories where discussed within the research group, resulting in a final coding tree (Figure S1). During this process, constant comparison was made with the raw data to ensure that the themes would cover all the data.

Further analysis was carried out in two stages. First, two researchers (AL, NW) focused on the coded data of all participants. Relationships between the main themes was discussed, resulting in a cross-thematic network, which was subsequently reviewed within the research group. Second, AL and NW compared and contrasted experiences in both the success and failure group, hereby re-evaluating the cross-thematic network within each group. Between-group differences in subthemes where described. The between-group differences in subthemes generated in the analysis were checked by frequency counts, which showed clear patterns matching those found in the interviews (Table 2)
