# Supplementary material for: Barriers and Facilitators Associated With App-Based Treatment for Female Urinary Incontinence: Mixed Methods Evaluation
Source: JMIR Mhealth Uhealth. 2021 Sep 17;9(9):e25878. doi: 10.2196/25878 (PMC8486988; doi:10.2196/25878)
Supplement: Supplementary file 3 [file mhealth_v9i9e25878_app3.docx]

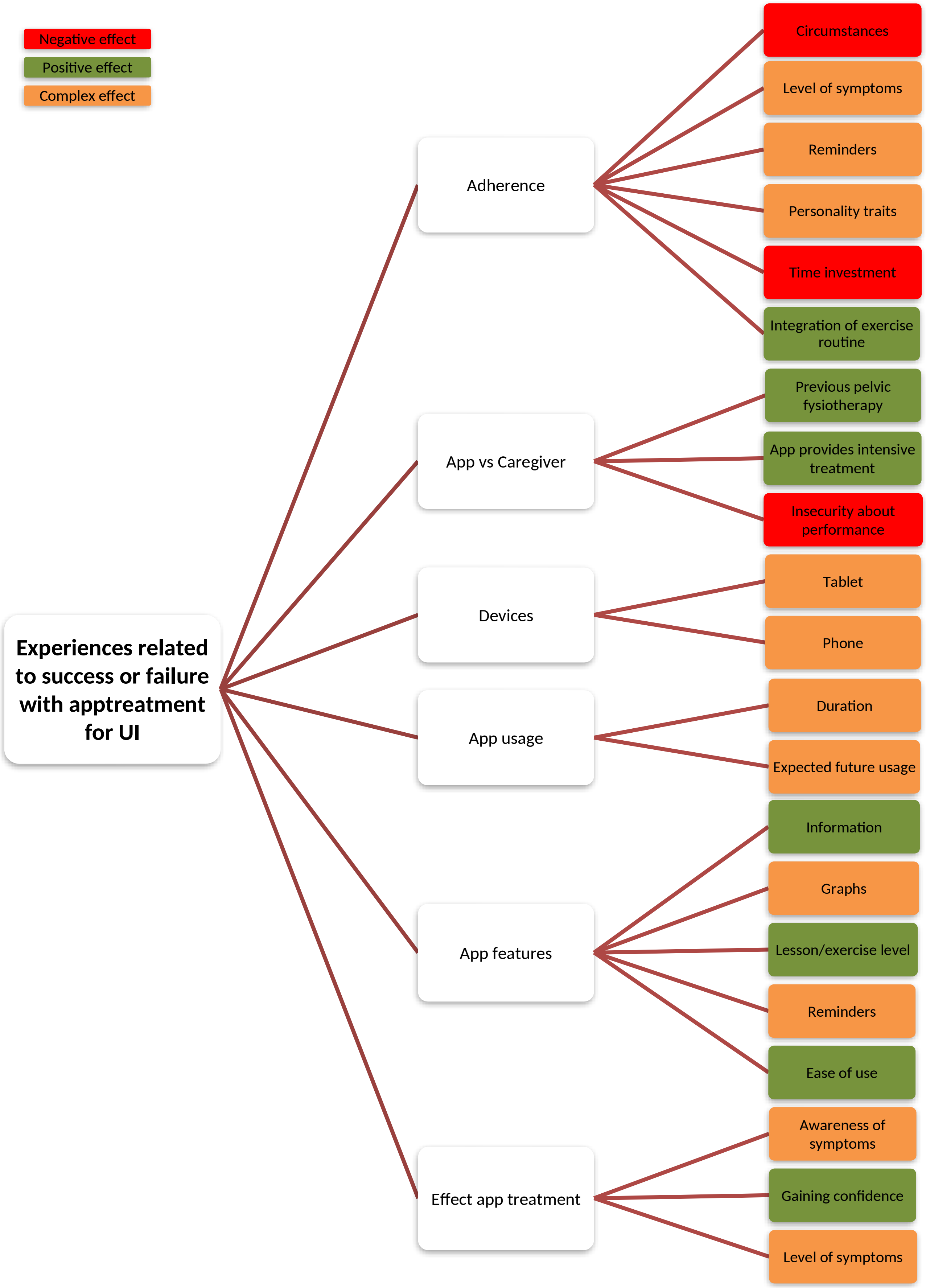

Negative effect
Positive effect
Complex effect
Experiences related to success or failure with apptreatment for UI
Adherence
Circumstances
Level of symptoms
Reminders
Personality traits
Time investment
Integration of exercise routine
App vs Caregiver
Previous pelvic fysiotherapy
App provides intensive treatment
Insecurity about performance
Devices
Tablet
Phone
App usage
Duration
Expected future usage
App features
Information
Graphs
Lesson/exercise level
Reminders
Ease of use
Effect app treatment
Awareness of symptoms
Gaining confidence
Level of symptoms
